# Lipid profiling analyses from mouse models and human infants

**DOI:** 10.1016/j.xpro.2022.101679

**Published:** 2022-09-15

**Authors:** Laurentya Olga, Ivana Bobeldijk-Pastorova, Richard C. Bas, Florine Seidel, Stuart G. Snowden, Samuel Furse, Ken K. Ong, Robert Kleemann, Albert Koulman

**Affiliations:** 1 Department of Paediatrics, University of Cambridge, Cambridge CB2 0QQ, UK; 2 Department of Metabolic Health Research, The Netherlands Organization for Applied Scientific Research (TNO), 2333 CK Leiden, the Netherlands; 3 DUCARES B.V. | trading as TRISKELION, Reactorweg 47-A, 3542 AD Utrecht, the Netherlands; 4 Core Metabolomics and Lipidomics Laboratory, Metabolic Research Laboratories, Institute of Metabolic Science, University of Cambridge, Cambridge Biomedical Campus, Cambridge CB2 0QQ, UK; 5 MRC Epidemiology Unit, Wellcome Trust-MRC Institute of Metabolic Science, NIHR Cambridge Comprehensive Biomedical Research Centre, Cambridge Biomedical Campus, University of Cambridge, Cambridge CB2 0SL, UK; 6 Institute of Metabolic Science, University of Cambridge, Cambridge CB2 0QQ, UK; 7 Department of Vascular Surgery, Leiden University Medical Center, 2333 ZA Leiden, the Netherlands

**Keywords:** Health sciences, Clinical protocol, Metabolism, Metabolomics, Mass spectrometry, Systems biology

## Abstract

This protocol outlines a translational lipidomic approach to discover lipid biomarkers that could predict morphometric body and histological organ measurements (e.g., weight and adiposity gains) during specific stages of life (e.g., early life). We describe procedures ranging from animal experimentation and histological analyses to downstream analytical steps through lipid profiling, both in mice and humans. This protocol represents a reliable and versatile approach to translate and validate candidate lipid biomarkers from animal models to a human cohort.

For complete details on the use and execution of this protocol, please refer to Olga et al. (2021).

## Highlights

• Development of translatable dyslipidemic/obese animal models to mimic human physiology
• Lipidomics in mice before/after exposure to high-fat diet to capture lipid biomarkers
• Validating lipid biomarkers from mice to human infants (reflecting early life context)
• Lipid profiling analysis on dried blood spots obtained from heel-prick in infants

## Abstract (editor highlights)

Publisher’s note: Undertaking any experimental protocol requires adherence to local institutional guidelines for laboratory safety and ethics.

## Graphical abstract

**Figure:**
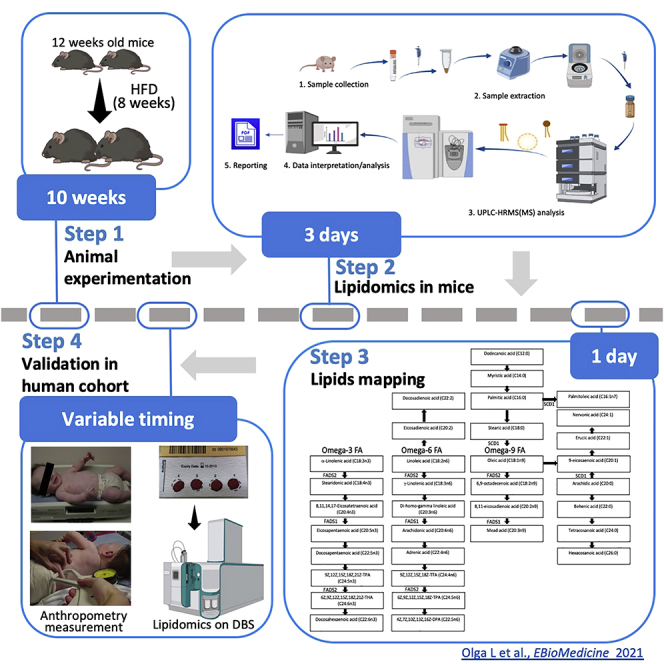

## Before you begin

### Induction of dyslipidemia and obesity in mice using translational approach

**Figure 1:**
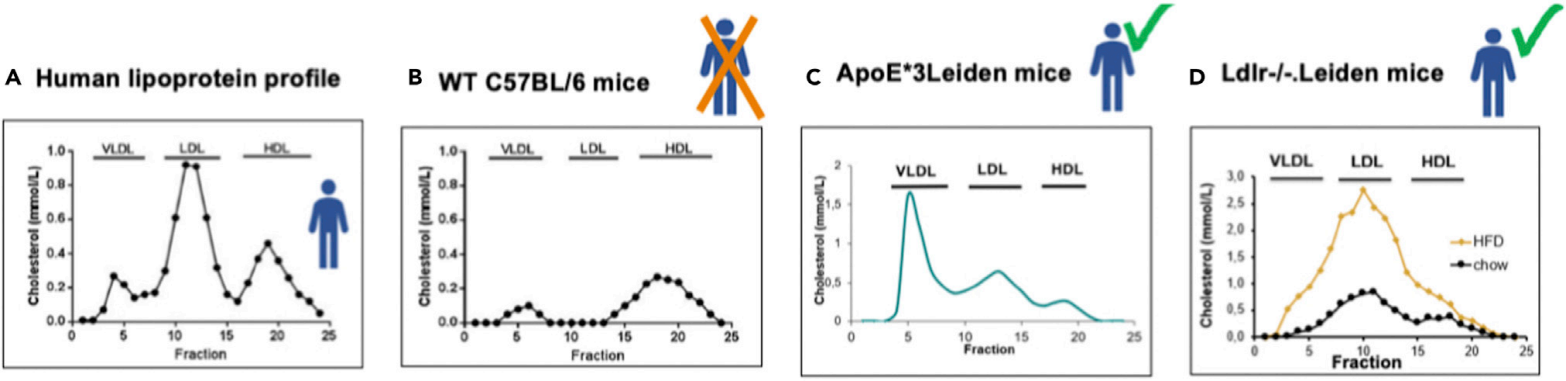
Suitable mouse strains for translational lipidomic experiments Illustration of the appropriate mouse strains to mimic human circulated lipoprotein profile that is dominated by VLDL/LDL particles. (A) Wildtype C57BL/6 is not appropriate since apolipoprotein B-containing particles (VLDL/LDL) in this strain are taken up by the liver and cleared from the circulation. (B and C) (B) Meanwhile, ApoE∗3Leiden (C) or Ldlr−/−. (D) Leiden mice (D) can be acceptable since their plasma lipids are confined to VLDL/LDL particles and levels can be increased with HFD. LDL=low-density lipoprotein, VLDL=very low-density lipoprotein, HFD=high-fat diets, WT=wild-type.

**Figure 2:**
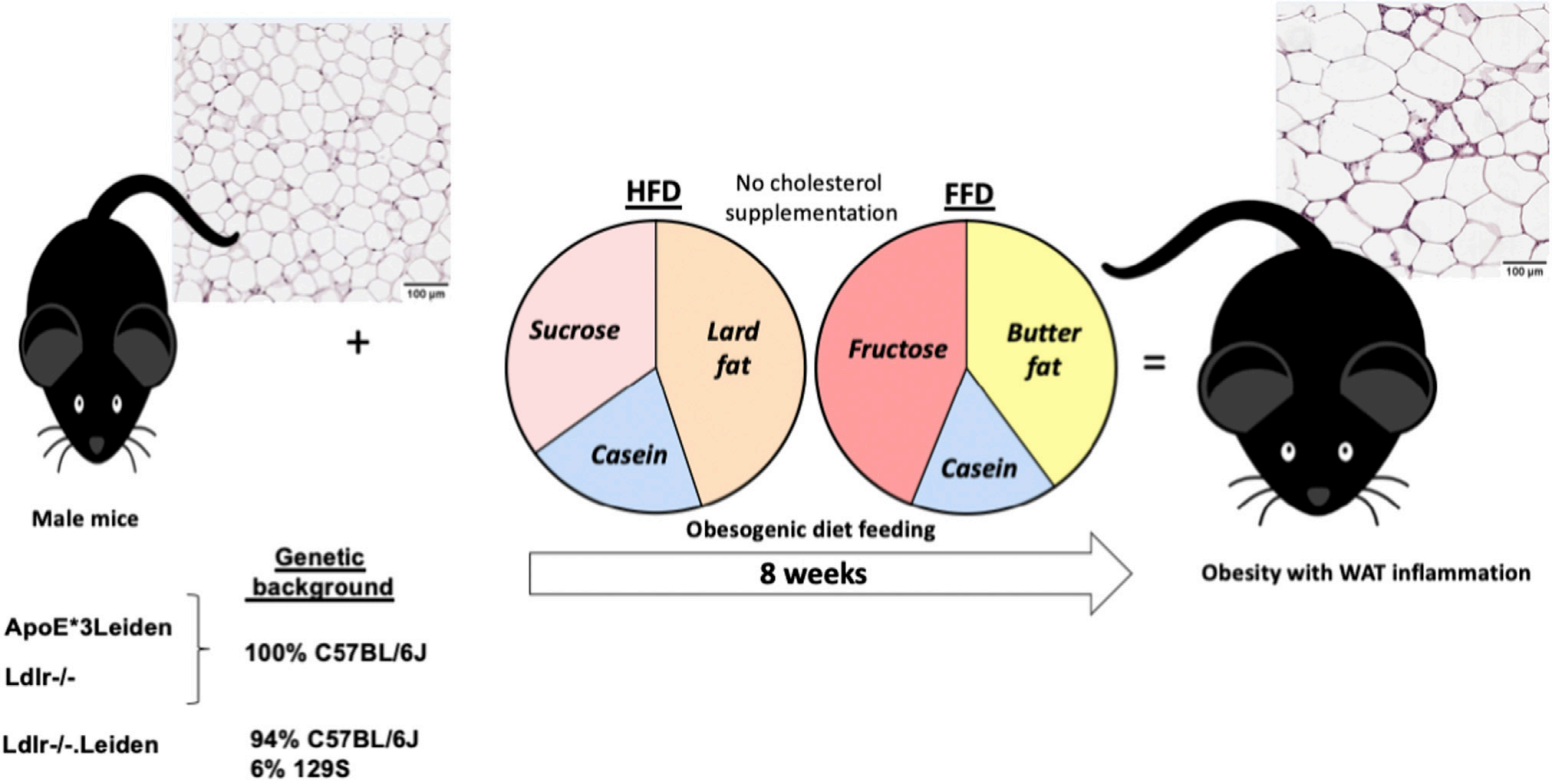
Obesogenic diets applicable for mice FFD=fast food diet, HFD=high-fat diet, WAT=white adipose tissue.

1. Select appropriate genetic background and type of diet, translatable from mice to humans.
  a. Breed or purchase mice (suggested N=30), predominantly from C57/BL6J genetic background as an inbred line with high predisposition to obesity and its associated inflammation.
    **CRITICAL:** Several genetic backgrounds are not susceptible to developing obesity (e.g., BALB/c mice) even when being fed with energy-dense high-fat diets (HFD). C57/BL6J is suitable for this purpose but researchers should familiarize themselves with its distinct metabolism and inflammation characteristics (Freeman et al., 2006; Ripoll et al., 2012; Nicholson et al., 2010).
    i. Feed animals with a low-fat rodent maintenance diet (9 kcal% fat, 33 kcal% protein, 58 kcal% carbohydrate; R/M-H Ssniff Spezialdiäten, Soest, Germany) prior to start of the experiment.
    ii. Do not start the experiment before sex hormones become stable (approximately at 10–12 weeks old).
      ***Note:*** Traditionally, it has been suggested to involve one gender only when conducting the experiment (preferably male; this experiment can actually be performed in any sexes, but male mice are inherently more vulnerable to develop pronounced obese phenotypes). Sex differences are however an important aspect of metabolism, describing and understanding of sex differences in model systems can help to interpret results of human cohorts.
      **CRITICAL:** Do not use a mixture of male and female mice due to sex-specific differences in fat mass, inflammatory tone, plasma lipids, and insulin metabolism (Jacobs et al., 2019). Besides, the general assumption that female (male) mice would better reflect women (men) is too simplistic and not supported by very basic genetic evidence since (1) Y chromosomes are remarkably divergent in structure and gene content among species (Hughes et al., 2010) and (2) there are striking species-specific differences with regard to X-inactivation in females (Sado and Sakaguchi, 2013).
  b. Select a mouse strain that is suitable for translational lipidomic research (Figure 1).
    ***Note:*** Examples of mice strains with established translational characteristics include ApoE∗3Leiden mice, ApoE∗3Leiden.CETP mice, Ldlr−/− mice or Ldlr−/−.Leiden mice (Zadelaar et al., 2007; van den Hoek et al., 2014; Morrison et al., 2018a, 2018b). These strains could develop human-like dyslipidemia characterized by high concentration of apolipoprotein B-containing particles (VLDL/LDL) when fed with HFD that contains macronutrient composition similar to human diet. Furthermore, apoE is required for lipase-mediated VLDL conversion to LDL (Medh et al., 2000), and the mutated human ApoE3 protein expressed in the transgenic ApoE∗3-strains would impair this lipase activity and cause VLDL to prevail in the circulation of these mice.
    **CRITICAL:** Wild-type and ob/ob mice are not suitable for this research because their predominant lipoprotein in the circulation is HDL. VLDL/LDL particles will undergo rapid hepatic clearance in this type of mice. Interestingly, ApoE−/− mice are also not suitable since they do not display adequate lipid alterations after HFD exposure (Schreyer et al., 2002).
    i. Accommodate mice for at least 2 weeks (preferably 4 weeks).
      ***Note:*** This is to reduce stress since stress might affect lipid metabolism and adiposity development.
    ii. House animals in social groups (n = 2–4) and keep cage groups intact.
      ***Note:*** In the case of fighting, split social groups in two cages but beware not to form new groups by mixing mice from different cages.
  c. Select and prepare relevant obesogenic diet (Figure 2).
    ***Note:*** Among animals, induction of obesity through diet relevant to humans requires moderate fat content of approximately 25% w/w or 45 kcal%. However, many standard HFD contain supraphysiological amounts of fat and therefore are not appropriate for animal-to-human translational studies. Furthermore, these experimental diets should contain approximately 20 kcal% of protein, with carbohydrate obtained from oatmeal, sucrose or fructose, and fibers (van den Hoek et al., 2020). This step of choosing a suitable mice diet to mimic human condition is important since among humans, a particular type of fat in the diet is more determining in increasing cardiovascular risk rather than the total amount of fat (Hu et al., 2001). The diet #D12451 (Research Diets, New Brunswick, NJ, USA) containing 45 kcal% fat from lard, 35 kcal% from sucrose, and 20 kcal% casein can be used, or fast-food diet as an alternative with 41 kcal% fat from milk fat, 44 kcal% from fructose and 14 kcal% casein.
    **CRITICAL:** Any energy-dense HFD supplemented with 0.2%–2% w/w cholesterol and most NAFLD/NASH-inducing diets are not suitable for translational studies because human diets contain much less cholesterol. Besides, cholesterol supplementation to a high-fat diet would rather rescue the desired dysmetabolism phenotype and counterintuitively improve insulin resistance resulting in comparable fasting plasma insulin levels as in normal chow diets as has been shown in time-course studies in obese C57BL/6 and ob/ob mice (Abe et al., 2019).

### Institutional permissions

All animal care, conditioning, and experiments should be performed in compliance with ARRIVE guidelines and the European Community specifications regarding the use of laboratory animals (or equivalent). All animal studies involved in establishing this protocol were approved by the Ethical Committee on Animal Care and Experimentation (Zeist, the Netherlands; approval reference numbers DEC-3117, -3076, -3260 and -3277).

Studies on humans in this protocol should comply with the Declaration of Helsinki. Participation of infants in the human infant cohort in this study was voluntary with informed written consent obtained from the mothers. This study was approved by National Research Ethics Service Cambridgeshire 2 Research Ethics Committee (REC) with REC reference 00/325.

## Key resources table

**Table:** 

REAGENT or RESOURCE	SOURCE	IDENTIFIER
**Chemicals, peptides, and recombinant proteins**		

2-Propanol ULC/MS - CC/SFC^a^	Biosolve	Cat#16264102
2-Propanol^c^	Sigma-Aldrich	Cat# 34863
Methanol Ultra LC-MS^a^	Actu-All Chemicals	Cat#813013802
MeOH (CAS: 67-56-1)	Sigma-Aldrich	Cat# 1.06007
Dichloromethane (stab. Amylene) AR	Biosolve	Cat#0013790501BS
Ammonia solution 25%	Merck	Cat#1.05432.1000
Ammonium acetate ULC/MS – CC/SFC	Biosolve	Cat#01244156
Water ultrapure	Merck	Milli-Q Reference
Tri-heptadecanoyl (C51:0 TG)^b^	Sigma-Aldrich	Cat#T2151
Cholesteryl heptadecanoate (C17:0 ChE)^b^	Sigma-Aldrich	Cat#C5384
Glyceryl tripalmitate (C48:0 TG)^b^	Sigma-Aldrich	Cat#T5888
Glyceryl tristearate (C54:0 TG)^b^	Sigma-Aldrich	Cat#T5016
Glyceryl trioleate (C54:3 TG)^b^	Sigma-Aldrich	Cat#T7140
Cholesteryl arachidonate (C20:4 ChE)^b^	Sigma-Aldrich	Cat#C8753
Cholesteryl linoleate (C18:2 ChE)^b^	Sigma-Aldrich	Cat#C0289
Cholesteryl stearate (C18:0 ChE)^b^	Sigma-Aldrich	Cat#C3549
Phosphate buffer saline (PBS)	Sigma-Aldrich Zwijndrecht, Netherlands	Cat# D8537
Hematoxylin solution	Sigma-Aldrich, Zwijndrecht, Netherlands	#MHS16
MTBE (CAS: 1634-04-4)	Sigma-Aldrich	Cat# 34875
1,2-di-O-octadecylsn-glycero-3-phosphocholine	Avanti Polar Lipids	Cat# 999991
1,2-di-O-phytanylsn-glycero-3-phosphoethanolamine	Avanti Polar Lipids	Cat# 999985
C8-ceramide	Avanti Polar Lipids	Cat# 860508
N-heptadecanoyl-D-erythro-sphingosylphosporylcholine	Avanti Polar Lipids	Cat# 860585
Undecanoic acid	Sigma-Aldrich	Cat# 89764
Trilaurin	Sigma-Aldrich	Cat# T4891
Ammonium acetate	Sigma-Aldrich	Cat# 73594

**Experimental models: Organisms/strains**		

Mouse: WT C57/BL6J (JAX®) (Age range: 8–10 weeks; Gender: Male)	Charles River, Sulzfeld, Germany	Strain code: 632 Coat color: Black
Mouse: ApoE∗3Leiden (Age range: 8–10 weeks; Gender: Male)	TNO, Leiden, Netherlands	Coat color: Black
Mouse: Ldlr−/−.Leiden (94% C57BL/6J and 6% 129S) (Age range: 8–10 weeks; Gender: Male)	TNO, Leiden, Netherlands	Coat color: Black

**Software and algorithms**		

GraphPad	Prism, San Diego, USA	Version 8.0.1 https://www.graphpad.com
Adiposoft	CIMA, University of Navarra, Spain	https://imagej.net/plugins/adiposoft This software was found to produce accurate results in comparison with manual analysis as the gold standard (Galarraga et al., 2012).
R	R Development Core Team	Version 3.6.0 https://cran.r-project.org/

**Other**		

Fast protein liquid chromatography (FPLC)	Pharmacia, Roosendaal, Netherlands	NA
Eppendorf tubes	Eppendorf, Hamburg, Germany	#0030123328
Slide scanner histology	Leica Biosystems, Amsterdam, Netherlands	Aperio AT2, Leica
Microtome	Leica Biosystems, Amsterdam, Netherlands	#RM2245/2255
Embedding cassettes	KLINIPATH, Duiven, The Netherlands	#410
Adhesive slides	KLINIPATH, Duiven, The Netherlands	#KP-SIL
Microcentrifuge	Thermo Fisher Scientific, Waltham, MA, USA	#75002555
Prefilled formaldehyde container	KLINIPATH BV, Duiven, The Netherlands	#4404-0200
Embedding paraffin	Leica Biosystems, Amsterdam, Netherlands	#39503002
Mounting Media	Leica Biosystems, Amsterdam, the Netherlands	#14070936261
LoBind microcentrifuge tubes 1.5 mL	Eppendorf	Cat#022431081
Autosampler vial 1.5 mL, 32 × 11.6 mm Amber Label	VWR	Cat#548-0030
Autosampler cap, combination seal 9 mm short thread	VWR	Cat#548-1533
Insert 300 μL for autosampler vial	No preference	NA
Vortex mixer	No preference	NA
pH meter	No preference	NA
Micro analytical balance	No preference	NA
Micro centrifuge for 1.5–2.0 mL tubes	No preference	NA
Ultrasonic bath	No preference	NA
Pipettes	No preference	NA
Acquity UPLC BEH Phenyl column, 1.7 μm, 2.1 × 100 mm	Waters	Cat#186002885
Q-Exactive Mass Spectrometer with HESI-II and APCI source	Thermo Scientific	NA
Dionex Ultimate 3000 RS UHPLC with pump, column compartment and autosampler	Thermo Scientific	NA
Pierce LTQ Velos ESI positive Ion calibration solution	Thermo Scientific	Cat#88323
2.4 mL deep well plate	Plate+TM	Cat# 60180-P338
220 μL deep well plate	Plate+TM	Cat# 60180-P333
Exactive (Benchtop Orbitrpa)	Thermo Fisher Scientific	SN01026P
Triversa Nanomate	Advion, Ithaca US	NA
96 head micro-dispenser (Hydra Matrix)	Thermo Fisher Scientific	NA
Genevac EZ-2 evaporator	Genevac Ltd.	NA

NA, not applicable.

a Used in animal study (plasma).

b Compounds of reference standard for lipidomics in plasma (mice study).

c Used in human study (dried blood spot).

## Step-by-step method details

### Animal study: Animal experiment and plasma collection

**Timing: Approximately 20 weeks**

This step describes HFD administration and blood sampling on mice, as well as plasma sample treatment and storage.

1. Regularly record food intake and weigh animals every 2 weeks.
2. Feed animals with HFD for the total period of 8 weeks, with 2 blood collection moments, each after a period of 4–5 h fasting. Blood should be collected:
  a. prior to commencing HFD.
  b. in the middle of HFD intervention, i.e., week 4.

***Note:*** The same fasting period is also applicable prior to sacrifice. It is necessary to standardize the fasting period during the entire study (e.g., consistently between 7-11 a.m.) to minimize diurnal lipid variation at blood collection.

3. Collect tail blood samples using EDTA-coated tubes (Sarstedt, Germany) according to local regulations on research using animals, preferably at baseline (before starting HFD or at the age of 12 weeks) and then every 4 weeks (at 16 and 20 weeks of age). For lipidomic research, around 10 μL plasma (∼30 μL of blood) is required. Store tubes on ice and prepare EDTA plasma within 30 min.

**CRITICAL:** If possible, avoid or limit the use of anesthetic agents prior to plasma collections since some of these compounds might affect plasma lipids.

4. Centrifuge those EDTA tubes at 4,000 rcf for 10 min at 4°C. Transfer out the supernatant (plasma) to Eppendorf tubes. Store at −70°C for subsequent lipidomic analyses.
5. The last EDTA plasma sample should be taken 1 day prior to sacrifice unless sufficient personnel are available to keep fasting plasma taken in strict and standardized procedure.

### Animal study: Study of WAT histology and inflammation

**Timing: Approximately 10 days**

This step elaborates on the mice sacrifice method as well as isolations and histological analyses of adipose tissue from different depots.

6. Weigh animals and sacrifice them within 2–3 h (or at the same time in two consecutive days in the case of limited personnel) according to the local ethical policy and animal welfare regulations (e.g., using isoflurane, CO/CO_2_ as appropriate).
7. Isolate organs of interest from cadavers, including epididymal white adipose tissue (WAT), inguinal subcutaneous WAT, and mesenteric WAT. Carefully weigh and record those different isolated depots of WAT.
8. Fix the isolated WAT depots in neutral buffered formalin (3.7% v/v formaldehyde) between 24–48 h.
9. Dehydrate the tissues by incubating them in increasing ethanol concentrations (e.g., 3 × 60 min 70%, 2 × 60 min 96%, 3 × 60 min 100%; v/v), then 2 × 60 min in 100% xylene, and finally 3 × 60 min in paraffin.
10. Embed the tissues in paraffin.
11. Prepare 5-μm cross-sectional cold paraffin blocks using a microtome cutting machine and place them in a drop of water on microscope glasses. Place the glasses on a heating plate (37°C) and when most of the water has been evaporated, transfer the glasses into a cell culture incubator (37°C) for further air-drying.

***Note:*** Air-drying is critical to prevent adipose tissue disruption which can occur due to too long incubation on the heating plate.

12. Deparaffinize the tissues with 2 × 2 min 100% xylene and rehydrate the cross-sections with decreasing ethanol concentrations (e.g., 1 min 50 s 100%, 10 s 96%, 10 s 70% v/v) and finally 3 × 5 min in 100% distilled water. Stain the cross-sections with hematoxylin-phloxine-saffron (HPS) following this procedure: 8 min Safran, 5 min hematoxylin, 3 min phloxine, and 20 s distilled water between each solution. Repeat tissue dehydration protocol: 70%, 96%, 100% ethanol for 20 s each and 2 × 3 min xylene, then coverslip them.
13. Take pictures at 20× magnification and perform a computer-assisted WAT cellularity (parameters: cell size and diameter as well as number of crown like structures), for instance using the open-source software Adiposoft (Galarraga et al., 2012).
14. To analyze tissue inflammation, randomly select 5 non-overlapping fields, distributed across the HPS-stained cross-sections. Afterward, quantify the number of crown-like structures (CLS), defined as circular structures formed by inflammatory cells around adipocytes.

### Animal study: Lipid profiling in mice

**Timing: Approximately 3 days**

This step explains lipidomic experiment and analysis in mice using ultra-performance liquid chromatography-high resolution mass spectrometry (UPLC-HRMS).

10 μL of plasma (sample) is extracted with 2-propanol containing internal standards (IS). The obtained extracts are analyzed for neutral lipids by UPLC-HRMS method using atmospheric pressure chemical ionization (APCI) in positive mode. In each series, an external calibration curve consisting of several representative compounds can be produced to quantify the compounds detected in the sample. Since standards are not available for every single compound detected in plasma, standards with the most structural resemblance are used in this protocol, assuming the response factors are equal. If desired, samples can also be analyzed relative to each other without any external calibrators. An estimate of the concentration of detected neutral lipid compounds can be made using IS. In this case, more IS should be used, preferably one for each class. The concentration of each IS should be adjusted to the range of expected concentration of the lipids. If specific lipid class of interest is composed of many lipids in very different concentrations, two IS per class can be used to estimate low and high concentration levels without exceeding the linear range of the detection.

***Note:*** Lipid extraction with organic solvents is usually the first step towards lipidomic analysis. However, standard method for lipid extraction from biological samples has not been established yet. The common practice usually involves phase separation between immiscible solvents, with lipids partitioned into hydrophobic phase i.e., traditional chloroform-methanol extraction. However, rather than employing this traditional method, this protocol uses an intermediate solvent (2-propanol) that has no phase separation to extract the neutral lipid fraction from plasma. This method has several advantages: 1) sample preparation is much faster and easier compared to the traditional method, 2) neutral lipids are recovered from the aqueous matrix (such as plasma), and 3) the solvents are inexpensive and less toxic.

***Note:*** High-performance liquid chromatography (HPLC) is commonly used in lipidomic analysis to separate lipids prior to mass analysis. Therefore, the vast majority of lipidomic studies use coupled and targeted liquid chromatography-mass spectrometry (LC-MS)/MS with electrospray ionization (ESI) in negative mode. However, since only neutral lipids are analyzed, this protocol employs untargeted UPLC-HRMS with APCI in positive ionization mode. The employed full-scan acquisition mode provides adequate sensitivity to semi-quantify the main neutral lipids present in plasma, i.e., mono, di-, and triglycerides, cholesterol, cholesterol-esters, and ceramides. Although LC-MS/MS.

***Note:*** The differences in the extraction step recoveries can be partially corrected by the use of lipid class-specific IS. But, due to different fatty acid chain lengths and saturation degree, such standards cannot fully account for different extraction behavior within lipid subclasses. Nevertheless, these differences are usually negligible in the extraction process.

15. Thaw samples at 20°C–22°C and homogenize
16. Prepare IS working solution (used for protein precipitation and lipids extraction from plasma) in 2-propanol containing two IS (C51:0 TG 2 μg/mL and C17:0 ChE 25 μg/mL).
  a. Make stock solution C51:0 TG (1 mg/mL).
    i. Weigh approximately 5 mg (accurate to 0.01 mg) of C51:0 TG in a 5 mL volumetric flask.
    ii. Add 500 μL dichloromethane and dissolve by ultrasonicating for 5 min.
    iii. Fill up to 5 mL with 2-propanol.
    iv. Shake the solution vigorously.
  b. Make stock solution C17:0 ChE (1 mg/mL).
    i. Weigh approximately 5 mg (accurate to 0.01 mg) of C51:0 ChE in a 5 mL volumetric flask.
    ii. Add 500 μL dichloromethane and dissolve by ultrasonicating for 5 min.
    iii. Fill up to 5 mL with 2-propanol.
    iv. Shake the solution vigorously.
  c. Make internal standards working solution C51:0 TG (2 μg/mL) and C17:0 ChE (10 μg/mL).
    i. Pipette 200 μL C51:0 TG stock solution and 1,000 μL C17:0 ChE stock solution into a 100 mL volumetric flask.
    ii. Fill up to 100 mL with 2-propanol.
    iii. Shake the solution vigorously.
17. Prepare mobile phases and needle wash solvent.
  a. Ammonium acetate solution (1 M).
    i. Weigh 7.7 grams of ammonium acetate in a 100 mL Schott Duran bottle.
    ii. Add 80 mL ultrapure water and dissolve by swinging the bottle.
    iii. Fill up to 100 mL with ultrapure water.
  b. Buffer solution (10 mM ammonium acetate with pH 7.0).
    i. Pipette 10 mL ammonium acetate solution (1 M) into a 1,000 mL Schott Duran bottle.
    ii. Fill up to 1,000 mL with ultrapure water.
    iii. Adjust to pH 7.0 by adding a few microliters of 25% ammonia.
  c. Mobile phase A (5% methanol in buffer).
    i. Add 950 mL buffer solution into a 1,000 mL Schott Duran bottle using a 1 L graduated cylinder.
    ii. Add 50 mL methanol using a 50 mL graduated cylinder.
  d. Mobile phase B (Methanol containing 2 mM ammonium acetate).
    i. Pipette 2 mL ammonium acetate solution (1 M) into a 1,000 mL Schott Duran bottle.
    ii. Fill up to 1,000 mL with methanol.
  e. Needle wash solvent (2-propanol:methanol = 60: 40 (^v^/_v_)).
    i. Add 600 mL 2-propanol into a 1,000 mL Schott Duran bottle using a 1 L graduated cylinder.
    ii. Add 400 mL methanol using a 1 L graduated cylinder.
18. Set up the calibration curve.
  a. Make a stock solution of C54:3 TG 500 μg/mL:
    i. Weigh approximately 12.5 mg (accurate to 0.01 mg) of C54:3 TG in a 25 mL volumetric flask.
    ii. Add 1,000 μL dichloromethane and dissolve by ultrasonicating for 5 min.
    iii. Fill up to the volume of 25 mL with 2-propanol.
    iv. Shake the solution vigorously.
  b. Make a calibration solution 6 containing C54:3 TG 25 μg/mL and C18:0 ChE 200 μg/mL.
    Weigh approximately 10 mg (accurate to 0.01 mg) of C18:0 ChE in a 50 mL volumetric flask.
    i. Add approximately 10 mL internal standards working solution and dissolve by ultrasonicating for 5 min.
    ii. Pipette 2.5 mL stock solution C54:3 TG into the volumetric flask.
    iii. Fill up to 50 mL with internal standards working solution.
    iv. Shake the solution vigorously.
  c. Make calibration solutions 0–5 following these steps:
    i. Pipet into a series of 25 mL volumetric flasks 0, 0.5, 1, 2.5, 5 and 10 mL of calibration solution 6, respectively (Table 1).
    ii. Fill up each volumetric flask to the volume of 25 mL with IS working solution.
    iii. Shake the solutions vigorously.
    iv. Transfer approximately 1 mL of each calibration solution into autosampler vials.
19. Prepare the samples:
  a. Pipette 10 μL of plasma samples into 1.5 mL Eppendorf tubes.
  b. Add 300 μL of IS working solution to the tubes.
  c. Vortex for 15 s.
  d. Centrifuge at 1,120 rcf for 5 min at 20°C–22°C.
  e. Transfer the clear supernatants into autosampler vials with compatible inserts and caps.

**Table 1:** Calibration solutions

Calibration solution^a^	C54:3 TG (μg/mL)	C18:0 ChE (μg/mL)
0	0	0
1	0.5	4
2	1	8
3	2.5	20
4	5	40
5	10	80

a In all calibration solutions the IS concentration for C51:0 TG is 2 μg/mL and for C17:0 ChE the concentration is 10 μg/mL.

***Note:*** Keep extracts at 20°C–22°C because triglycerides will precipitate at low temperatures. For long term storage (>2 days), store extracts in the freezer (< −18°C).

20. Prepare quality controls:
  ***Note:*** Quality controls are preferably pooled from the same study. If this is not feasible, quality controls can be obtained from the same type of sample (e.g., plasma) and ideally from the same species (in this case should be from the same mouse model) available on stock will be used for quality control.
  Two types of quality control samples will be prepared: A and B.
  A is QC sample for monitoring the system stability and therefore a larger volume of QC A sample will be extracted, aliquoted into vials, and stored at < −18°C until analysis. QC A will be added to each analyzed batch of sample, minimum 2 times, see also Sequence.
  B is QC sample for monitoring batch-to-batch differences during sample preparation. These samples will be aliquoted before the extraction and stored at < −18°C. A minimum of 2 QC B samples will be added to each batch of samples, see also Sequence. More QC B samples are preferred and are distributed evenly throughout the batch before sample extraction step.
21. Prepare experimentation sequence:

Samples randomization or division into batches might differ from project to project. This order can be followed:

Blank solvent.

Calibration curve of the standard solutions (Calibration solutions 0–5, Table 1).

Blank solvent.

QC A.

(Sample 1–10).

QC B.

(Sample 11–20).

Repeat min 5×.

QC A.

Blank solvent.

Calibration curve of the standard solutions (Calibration solutions 0–5, Table 1).

22. UPLC-HRMS analysis.

Neutral lipids are separated on a Waters Acquity BEH phenyl column (100 × 2.1 mm, 1.7 μm; T = 60°C) using a mobile phase gradient from 40% mobile *phase* A [5% methanol in 10 mM NH_4_Ac] to 100% mobile *phase* B (100% methanol containing 2 mM NH_4_Ac) in 13 min with a flow of 0.4 mL/min. Mass detection is carried out using atmospheric pressure chemical ionization (APCI) in the positive mode. Injection volume is 2 μL.

The ultra-performance liquid chromatography high resolution mass spectrometry (UHPLC-HRMS) system consists of a Dionex Ultimate 3000 UHPLC system coupled on-line in series to a quadrupole-orbitrap mass spectrometer (Q-Exactive, Thermo Electron Corporation, Bremen, Germany). The equipment is controlled using Thermo Fisher Scientific Xcalibur™ v 4.1 Foundation 3.1 SP4 software.

Data processing is performed using XCalibur software (v4.1, Thermo Fisher Scientific) with target lipids and their accurate masses listed in Table S1.

### Lipid mapping onto lipid metabolism pathways

**Timing: Approximately 1 day**

This step describes the mapping of lipid species captured from animal experiments onto common lipid metabolism pathways shared across species.

**Figure 3:**
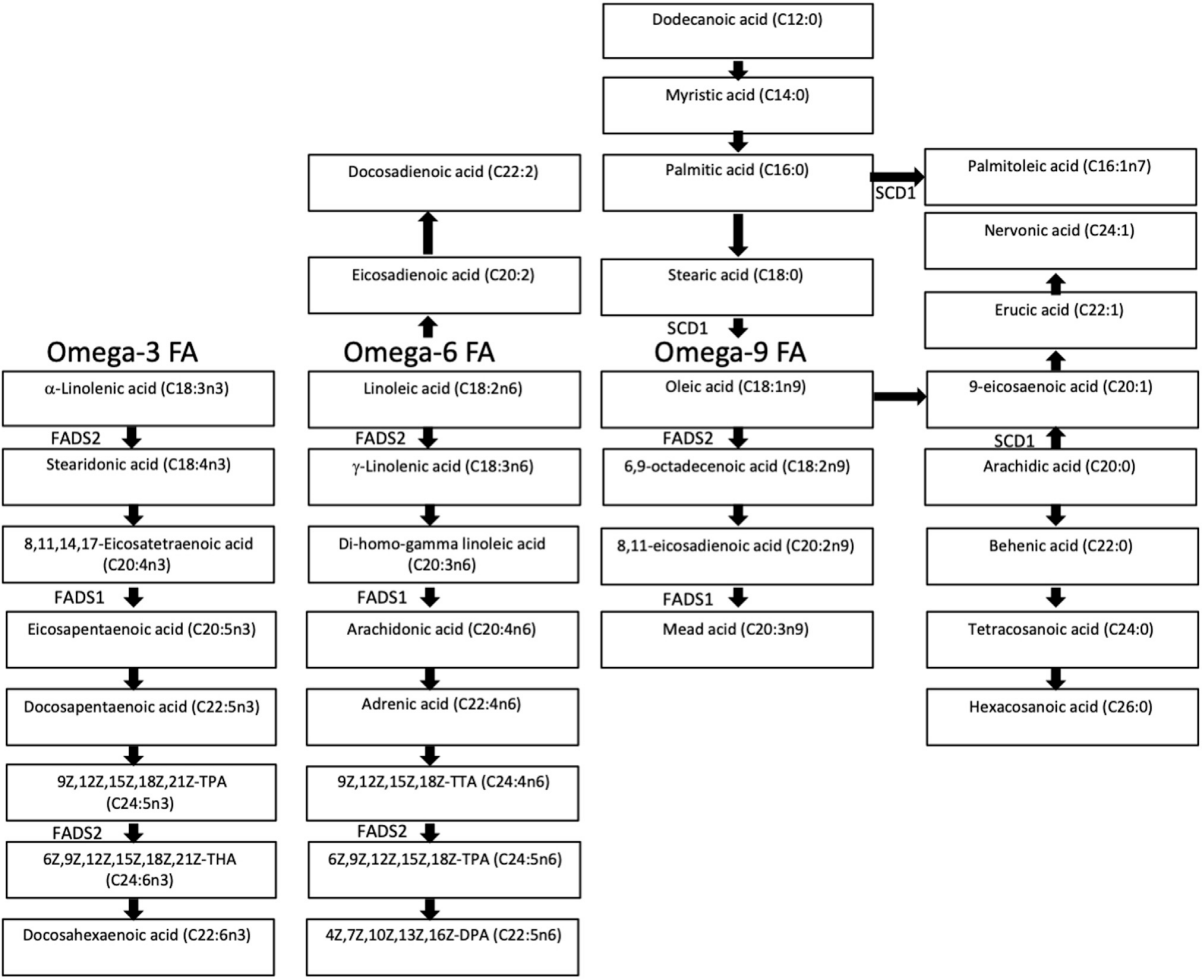
Lipid metabolism pathways

***Note:*** Although lipids and lipid metabolism differ across species, many lipid metabolism processes are evolutionarily conserved and thus could be still comparable between species, e.g., de novo lipogenesis, hepatic triacylglycerols formation, or fatty acid catabolism. Of note, species differences should be still considered carefully, especially regarding the activity and allosteric regulation of the enzymes involved. The lipid moieties, however, are still comparable and the conversion of omega-3, omega-6 and omega-9 fatty acids depicted in the desaturase framework in Figure 3 (Olga et al., 2021) apply to both human and mouse fatty acids.

***Note:*** Since the fatty acid conversions that are enzymatically catalyzed by desaturases (Figure 3) occur in both humans and mice, ratio of the fatty acid used as substrate (educt) and the resulting fatty acid product that is formed provides a measure for desaturase activity (i.e., desaturase activity index). As desaturases catalyze several reactions and use several fatty acids as substrate, desaturase activity index of enzyme can be calculated in several different ways.

***Note:*** Desaturase activity indexes should ideally be calculated from the same lipid class. However, if translational studies are conducted using different methods, alternative ratios/calculations can be employed. For instance, in our study (Olga et al., 2021); since mouse and human lipidomics were using different platforms and human sample was obtained through the least invasive method possible (i.e., heel prick among infants) resulting in limited amount of sample in dried blood spots, SCD-1 activity index was calculated from cholesteryl esters in mice and from both cholesteryl esters and phosphatidylcholines in humans.

23. Map the candidate lipid biomarkers found from the animal study onto lipid metabolism pathways (Figure 3).

### Human study in an established infant cohort

After mapping the candidate lipid biomarkers onto the corresponding lipid metabolism pathways, the next step is to validate these findings in a longitudinal infant cohort. However, there are practical considerations that limit such investigative studies of metabolic programming in infants, mainly due to ethics and difficult sample collections in small infants. Therefore, although plasma is the ideal sample for lipidomic experiments, dried blood spot obtained from heel-prick is more preferable in infants since the method is less invasive and thus can increase compliance and number of samples. Moreover, this method has also been validated and is evidenced to be able to provide sufficient materials for lipidomic analyses (Koulman et al., 2014; Prentice et al., 2015; Acharjee et al., 2017).

### Human study: Longitudinal anthropometry measurement

**Timing: throughout the study period; each time of physical measurement takes about ∼90 min**

Since the outcome of the study is weight/adiposity gain, in particular during early life, this step sets out the conduction of a prospective infant human cohort with multiple visits to measure longitudinal growth and body composition parameters, including weight, height, and skinfold thicknesses (SFT).

***Note:*** Study design must be thoroughly explained to potential study participants before informed consent to minimize the possibility of dropping out before completing the study.

Infant anthropometry measurement should be performed by pediatric research nurses, properly trained by an anthropometry expert.

To ensure the quality of data produced:

i. Each infant clinic visit should be scheduled based on the exact age of infants with +7 days tolerance for birth, 2-, and 6-weeks visits, and +28 days for 3 months onward. If possible, record birth weight from the medical/hospital records.
ii. Regular machine calibration and personnel training should be conducted periodically.
iii. Data cleaning has to be applied in all anthropometric measurements prior to statistical analyses to exclude any implausible and invalid data values (e.g., 0.37 kg instead of 3.7 kg of birth weight).

24. Measure infant weight to the nearest 1 g using Seca 757 electronic baby scale (Hamburg, Germany).

***Note:*** Infants are weighed nude without nappies, or alternatively the weight of the diaper can be subtracted from the resulting weight. Weight measurement should be made before feeding.

25. Measure infant supine length to the nearest 0.1 cm using Seca 416 infantometer (Hamburg, Germany).

***Note:*** Standing height was measured using a stadiometer if subjects could stand without assistance, typically from 2 years old onwards.

**CRITICAL:** Measurement of length/height should be strictly performed without foot- and headwear.

26. Use weight and length/height measurements to calculate body mass index (BMI) by dividing weight by the square of length/height.
27. Measure SFT at multiple sites in triplicate on the left-hand side of the body using a Holtain Tanner/Whitehouse Skinfold Caliper (Holtain Ltd, Crymych, Wales, UK), e.g., in 4 sites: triceps (at the posterior surface of the arm, halfway between the acromial process or shoulder and the olecranon or elbow), subscapular (at the oblique angle below the scapula or upper back), flank (in the posterior axillary line immediately posterior to the iliac crest), and quadriceps (in the midline and halfway between the top of the patella and the inguinal crease).

***Note:*** SFT reflect subcutaneous fat folds and can be used to assess subcutaneous fat at various regions of the body or summed to estimate relative total body fatness (Diet, Anthropometry and Physical Activity (DAPA) Measurement Toolkit, 2019).

### Human study: Dried blood spot collection in infants

**Timing: throughout the study period; each time of sample collection requires ∼30 min**

This step focuses on collecting, treating, and storing dried blood spots obtained from heel prick as the least invasive blood sampling method in infants.

28. Perform heel-prick in infants and record their last feed.

***Note:*** Since fasting prior to sample collection can be considered unethical for an infant study, recording the time and type of last feed is critical. These data can then be covariates to adjust for in data analyses.

29. Drop bloods onto Whatman™ 903® untreated filter paper (Ahlstrom 226, ID Biological Systems), similar to the method employed in the UK newborn routine screening program.

***Note:*** Ensure that the blood goes through the back of the paper to maximize the amount of captured biological materials.

30. Air-dry the samples at 20°C–22°C for 16–24 h.
31. Store the samples at −20°C or −80°C (preferably −80°C).

***Note:*** Use uniform temperature throughout to avoid results dissimilarities due to temperature differences.

32. At the time of lipid profiling analysis, punch out a single aliquot spot with diameter ∼3.2 mm from the larger DBS spots.

### Human study: Lipid profiling in human samples

**Timing: Approximately 5 days, depending on the number of samples**

This step describes the whole process of lipid profiling analysis from human dried blood spots and the steps of lipidomic data processing.

Each experiment should be conducted with blank controls, 2 different quality control samples (mixed anonymized adult human venous blood samples and commercially available horse blood), and 6 IS (0.6 μM 1,2-Di-O-octadecyl-*sn*-glycero-3-phosphocoline, 1,2 μM 1,2-di-O-phytanyl-*sn*-glycero-3-phosphoethanolamine, 0.6 μM C8-ceramide, 0.6 μM *N*-heptadecanoyl-D-erythro-sphingosylphosphorylcholine, 6.2 μM undecanoic acid, 0.6 μM trilaurin). The experiment begins by eluting the punched-out 3.2 mm DBS in methanol containing those 6 IS in 1.2 mL cryovials. This process results in lipids being partitioned into methyl tertiary butyl ether. Then, samples are centrifuged at 4,000 rcf for 10 min to get the organic layer (that will be used for lipid analysis) separated. Afterward, infuse samples into a Thermo Exactive benchtop orbitrap (Hemel Hampstead UK) using an Advion Triversa Nanomate (Ithaca US) and acquire data from both positive (+1.2 kV) and negative (-1.5 kV) modes. Data obtained from this procedure will be in semi-quantitative form or as signal intensity of each lipid expressed relative to the total lipid signal acquired for each individual per thousand (^0^/_00_).

33. Lipids extraction.
  ***Note:*** The automated method for the extraction of lipids is developed using an Anachem Flexus automated liquid handler (Anachem, Milton Keynes, UK).
  a. Place in total 80 dried blood spot samples, 4 blanks, 8 QC1 and 4 QC2 as 3.2 mm diameter discs in 1.2 mL Cryovials, which shall then be placed in the wells of a glass-coated 2.4 mL deep well plate (Plate+™, ESSLAB, Hadleigh, UK).
  b. Add 100 μL of MilliQ H_2_O to each of the wells.
  c. Add 250 μL of MeOH containing six IS (0.6 μM 1,2-di-O-octadecylsn-glycero-3-phosphocholine, 1.2 μM 1,2-di-O-phytanylsn-glycero-3-phosphoethanolamine, 0.6 μM C8-ceramide, 0.6 μM *N*-heptadecanoyl-D-erythro-sphingosylphosporylcholine, 6.2 μM undecanoic acid, 0.6 μM trilaurin), followed by 500 μL of methyl *tert*-butyl ether (MTBE).
  d. Seal the plates (using Corning aluminum microplate sealing tape Sigma Aldrich Company, UK) and shake for 10 min at 40 rcf; after this, transfer the plate to a centrifuge and spin for 10 min at 4,000 rcf.
  e. Each well in the resulting plate shall now have two layers, an aqueous layer at the bottom and an organic layer on top.
  f. Using a 96 head micro-dispenser (Hydra Matrix, Thermo Fisher Scientific Ltd., Hemel Hampstead, UK) transfer 200 μL of the organic layer to a glass-coated 240 μL low well plate (Plate+™, ESSLAB, Hadleigh, UK).
  g. Transfer the plate to a Genevac EZ-2 evaporator (Genevac Ltd., Ipswich, UK) and dry down.
  h. The samples shall then be reconstituted in 25 μL of MTBE and with 90 μL of MS-mix [7.5 mM NH_4_Ac IPA: MeOH (2:1)] using a Hydra Matrix, after this the plate can be sealed and stored at −20°C until analysis.
34. Mass spectrometry.
  ***Note:*** Direct infusion high resolution MS (DIHRMS) is required for this experiment.
  a. Using a Triversa Nanomate (Advion, Ithaca US), all samples shall be infused into an Exactive Orbitrap (Thermo Fisher Scientific, Hemel Hampstead, UK).
    ***Note:*** The Nanomate infusion mandrel shall be used to pierce the seal of each well before analysis, after which, with a fresh tip, aspire 5 μL of the sample followed by an air gap (1.5 μL).
  b. The tip shall then press against a fresh nozzle and the sample will be dispensed using 0.2 psi nitrogen pressure; Ionization can be obtained with a 1.2 kV voltage.
  c. The Exactive shall start acquiring data 20 s after sample aspiration begins.
  d. After 72 s of acquisition in positive mode, the Nanomate and the Exactive shall switch over to the negative mode, decreasing the voltage to -1.5 kV.
  e. The spray shall maintain for further 66 s, after which the analysis shall be stopped and the tip shall be discarded, before the analysis of the next sample begins.
  f. Throughout the analysis the sample plate will be kept at 15°C.
  g. All the samples shall continue to run in row order.
35. Lipidomic data conversion and processing.
  a. Convert the raw data to “.mzXML” (MS convert) file format, then parse to R (with a 50 spectra average per mode) using XCMS.
  b. Process the data further using Peak-picker v2.0 and an in-house R script for peak picking, de-isotoping and annotating DIHRMS data sets (R-codes are available upon request).
  c. All significant thresholds can be set using the Bonferroni multi-comparison correction; Multivariate analysis can then be performed in SIMCA (v13.0) (Umetrics) or in online resources such as Metaboanalyst (www.metaboanalyst.ca).

### Statistical analyses

**Timing: Approximately 5 days, depending on the research questions and the number of samples**

This step describes the statistical analysis strategy to analyze lipidomic data harvested in this protocol, including correlation analysis in the animal study and multiple linear regressions in the human study.

36. Statistical analyses in animal study.
  a. Examine the correlations between plasma lipids variation (due to exposure to HFD) and obesity endpoints using Spearman’s tests.
  b. Apply p-values corrections for multiple testing by calculating the false discovery rate (FDR) using Benjamini Hochberg method.
37. Statistical analysis in human study.
  a. Derive age- and sex-appropriate standard deviation scores (SDS) for weight and length/height by comparison to relevant standard/reference, e.g., WHO, CDC, or UK 1990, using LMSgrowth software (Pan and Cole, 2012). Further adjustment for gestational age is recommended for measurements taken at 0–3 months.
  b. Derive internal SDS for each individual SFT using the whole cohort population by running multiple linear regression adjusted for infant sex, gestational age, and exact postnatal age at visit and saving the residuals. Calculate mean SFT SDS at each time point.
  c. Growth gains can be calculated from measurement delta between 2 time points, e.g., 3–12 and 12–24 months.
  d. Analyze the associations between lipids of interest with growth gains using multiple linear regressions, adjusted for any relevant and recorded maternal, perinatal, and infant factors, e.g., maternal age, pre-pregnancy BMI, parity, mode of delivery, pregnancy/neonatal comorbidities, infant feeding history, etc.

***Note:*** Apart from multiple linear regressions, multivariate analyses including principle component analysis (PCA) or partial least squares-determinant analysis (PLS-DA) can also be used when analyzing lipidomic results.

38. Univariate analyses for animal study can be performed in Prism 6.01 or above (GraphPad Software, Inc.). Analyses for human study can be conducted using SPSS 25.0 or above (IBM) or R version 1.0.3 or above.

## Expected outcomes

Lipidomic analysis using human biological samples may produce inconsistent results, typically due to observational study design, and therefore is limited to study metabolic/mechanistic pathways. On the contrary, animal experimentation can offer more conclusive results since it may allow strict diet intervention, uniformize genetic as well as environmental settings, and enable thorough tissue investigation. However, lipidomic results from animals still need translation to humans since lipid metabolism is unique across species and nutrient density per body weight is different between humans and mice (Innis, 2000).

From our mice experiments, several lipids (mainly ChE and PC) were found to correlate with obesity parameters, including weight gain, after 8 weeks of extreme diet or HFD among transgenic mice (E3L or Ldlr−/−), but not among wildtype C57BL/6J. Four ChE with strongest correlation coefficients with subsequent weight gain were then mapped onto lipid metabolism pathways and were shown to be educts or products of desaturases activities. Using lipid ratios as proxies of estimated desaturases activities, we observed associations between those desaturases, SCD1, FADS1, and FADS2, with subsequent growth and body composition parameters among infants, suggesting them to be potential lipid biomarkers to predict weight gain during early life (Olga et al., 2021).

## Limitations

Histology analysis of adipocytes in mice may possess technical limitation due to difficult exact assessment of cell size and diameter. Adipocytes are arranged in a three-dimensional network and when histological cross-sections are prepared, most adipocytes (even the fully round ones) are not exactly cut in their middle. Consequently, the quantified adipose tissue cellularity readouts (e.g., its diameter) represents an average value from the analysis of hundreds of cells. Therefore, this average value of adipocyte diameter should not be interpreted as an absolute diameter of adipocytes in any particular adipose tissue depot.

The lipidomic analytical methods employed in this protocol aim to capture the most abundant lipids and therefore if specific signaling lipids, e.g., oxylipins, are of interest, different sample preparation and analysis methods should be used.

Since different analytical conditions and mouse strains may affect the outcomes of the experiment, understanding their characteristics and mechanistic basis is of utmost importance. As shown in Figure 1 (A vs B), humans and mice have different lipoprotein profiles. Unlike in humans, lipoprotein particles in mice are rapidly cleared from circulation. Therefore, to mimic human lipoprotein profile, hepatic clearance reduction is necessary, e.g., by knocking out LDL receptor, deleting apoE, or increasing the expression of human genes that can compete with apoE to bind at the LDL receptor (Zadelaar et al., 2007; Dewey et al., 2017). Acknowledging this, the use of wildtype C57BL/6J mice strain in this protocol might actually be comparable to the other strains, e.g., C57BL/6N, but it has been examined formally by the authors.

Lipid transfer in the plasma compartment is also different between humans and mice due to the absence of cholesteryl ester transfer protein (CETP) in all mouse strains. In humans, CETP plays a crucial role by exchanging lipids between lipoproteins, i.e., transferring triglycerides from apolipoprotein B-containing particles (VLDL/LDL) to HDL in exchange for cholesteryl esters. This makes lipid fluxes in humans more complicated but it should not affect the validity nor the importance of the biomarkers described in this protocol since our lipidomic approach quantifies the entirety of all cholesteryl esters, irrespective of being parts of VLDL/LDL and HDL particles or not.

Specifically in the human/infant experiment, due to the spotting and drying process used to obtain DBS samples, several lipid species will be inevitably oxidized, mainly unsaturated fatty acid-containing lipids across all lipid classes. Therefore, signals from these lipid species should not be used in further analyses. Any lipid species where more than 30% of subjects had a value of zero should also be excluded.

Lastly, the desaturases are enzymes that reside in tissues and their activity in different human tissues has thus far not been analyzed. It is therefore not known whether desaturase indexes determined from plasma lipid ratios (e.g., C18:1/C18:0) reflect the desaturase activity of the liver or another tissue, i.e., a particular adipose tissue depot, or the combination of both. To shed a light on this, human studies will be necessary in which biopsies from multiple organs will be collected together with corresponding blood samples.

## Troubleshooting

### Problem 1

It is possible that the mice that are housed in a cage start to fight during the night. This may result in wounds which may not be noticed easily.

### Potential solution

Monitor mice daily and inspect their skin to investigate any presumed stress. One mouse could be superior to the others and therefore it is possible to separate the mice and house them in separate cages. Since mice are social animals, try to keep several mice together and minimize single housing. Diluted lavender essential oil can also be spread in the cages as an alternative. To treat the wounds, apply wound gel regularly until they are healed.

### Problem 2

Mice do not gain weight as expected (compared to the other studies) or adipose tissue inflammation does not develop.

### Potential solution

Inadequate body weight gain is often related to stress from noise, disturbances, smells (e.g., sacrifice is performed in the adjacent rooms), and frequent or inadequate animal handling. If external causes can be excluded, check the exact background of the mice and the sources of macronutrients in the diet: ensure that beef tallow is not the source of fat, sucrose/fructose is not the source of carbohydrate, and type of protein does not include casein hydrolysate (it should be intact casein instead).

### Problem 3

Suboptimal bleeding occurred during blood collection resulting in low plasma volume.

### Potential solution

Dilating tail vein to obtain blood could be ineffective in some conditions, e.g., if the body temperature is low. To solve this, mouse can be placed under infrared light or other heating lamps. In addition, its tail can be massaged with light pressure from the body towards its top. This should be done carefully because it can lead to hemolysis, i.e., rupture of the blood cells and release of their content into the plasma.

### Problem 4

Plasma readouts (in particular lipids) are not homogenous and animals appear to have consumed food although they were fasted.

### Potential solution

The mice might have eaten crumbles of the diet and/or feces that was present in the cages. When mice are subjected to fasting, do not just remove diet from the trays, but transfer mice into cages with fresh bedding instead.

### Problem 5

During adipose tissue histological analysis, when preparing cross-sections from the paraffine blocks, the obtained sections might be striated or torn.

### Potential solution

Verify that the knife is sharp and change it if necessary. The cooling plate might also be too cold; it should not be set below - 10°C. If all these adjustments have been made, use another paraffine-embedded tissue, e.g., liver, that is easier to handle and prepare cross-sections from this tissue prior to continuing with adipose tissue. The time needed to fix adipose tissue with formaldehyde may also be prolonged up to one week, depending on the size of biopsies. If entire adipose tissue depots are incubated in formaldehyde, a fixation period of about 7 days is recommended.

### Problem 6

During adipose tissue histological analysis, paraffine ribbon becomes static and therefore cannot be handled or placed at a desired spot.

### Potential solution

Use wet wipes to move over paraffine gently prior to cross-sectioning. Of note, the tissue should only be slightly wet (moist, not soaked with water) to avoid obtaining thicker cross-sections.

### Problem 7

Weak signal captured from MS.

### Potential solution

Check if these problems present and fix accordingly: incorrect injection (wrong volume, leaking injector, clogged injection needle), incorrect samples, suppression in the MS, incorrect calibration, incorrect instrument settings.

### Problem 8

Shifting retention times when performing lipidomics using MS.

### Potential solution

Check if these problems present and fix accordingly: incorrect flow rate or flow rate is reduced by a leak, inadequate degassing mobile phase or incorrect mobile phase, faulty check valves and/or pump seal failure, HPLC column contamination and fouling, incorrect column oven temperature, no stable ESI spray with MS.

### Problem 9

Infants do not bleed sufficiently through heel-prick, resulting in no gone-through blood spots in the filtered paper.

### Potential solution

Make sure that the foot is warm enough and gently massage it prior to performing heel-prick, aiming for vein dilation.

### Problem 10

In contrast to problem 1, infant bleeds excessively or difficult to stop bleeding.

### Potential solution

Parents can be distressed when seeing their infants bleeding excessively and this can cause a drop out of the study. This is important to prevent since retention rate is vital in longitudinal study. Make sure that the infant does not have any blood coagulation problems by always checking clinical notes prior to visit. Excessive bleeding can be caused by stress or crying so feed the baby while performing heel-prick could usually distract the baby’s attention, while also provide comfort and reduce the pain caused by it.

### Problem 11

Weak lipid signals to capture.

### Potential solution

Dried blood spots are not the most ideal samples to provide in lipidomic study due to high risk of oxidation and low amount of sample compared to plasma or serum. However, it is important to perform the least invasive method of blood collection in an infant cohort, i.e., heel-prick. Therefore, strict sample handling could help to maximize the use of dried blood spot. Make sure to obtain blood spots that go through the filtered paper and air-dry the filtered cards for 24 h. Afterward, store the cards well in the freezer, preferably at −80°C.

## Resource availability

### Lead contact

Further information and requests for resources and reagents should be directed to and will be fulfilled by the lead contact, Dr Albert Koulman (ak675@medschl.cam.ac.uk).

### Materials availability

There are no newly generated materials generated from this study.

## Data Availability

The datasets supporting the current study have not been deposited in a public repository but are available from the corresponding author on request.
