# Supplementary material for: Lipid profiling analyses from mouse models and human infants
Source: STAR Protoc. 2022 Sep 15;3(4):101679. doi: 10.1016/j.xpro.2022.101679 (PMC9486117; doi:10.1016/j.xpro.2022.101679)
Supplement: Document S1. Table S1 [file mmc1.pdf]

Table S1. Target lipids and their accurate masses, related to Step 23

Diglyceride	m/z $[M+NH_4]^+$	Triglyceride	m/z $[M+NH_4]^+$	Cholesterol ester	m/z $[M+NH_4]^+$
C32:0 DG	586.5420	C48:0 TG	824.7729	C16:0 ChE	638.5860
C34:2 DG	610.5420	C50:0 TG	852.8043	C16:1 ChE	640.6017
C34:3 DG	608.5263	C50:1 TG	850.7886	C16:2 ChE	638.5883
C36:2 DG	638.5734	C50:2 TG	848.7729	C17:0 ChE	656.6354
C36:3 DG	636.5577	C50:3 TG	846.7572	C18:0 ChE	670.6511
C36:4 DG	634.5420	C50:4 TG	844.7415	C18:1 ChE	668.6354
C36:5 DG	632.5263	C50:5 TG	842.7258	C18:2 ChE	666.6197
C38:2 DG	666.6048	C51:0 TG	866.8200	C18:3 ChE	664.6040
C38:3 DG	664.5891	C52:0 TG	880.8357	C20:1 ChE	696.6668
C38:5 DG	660.5577	C52:1 TG	878.8200	C20:2 ChE	694.6511
C38:6 DG	658.5420	C52:2 TG	876.8043	C20:3 ChE	692.6354
C38:7 DG	656.5263	C52:3 TG	874.7886	C20:4 ChE	690.6197
C40:6 DG	686.5734	C52:4 TG	872.7729	C20:5 ChE	688.6040
C40:8 DG	682.5420	C52:5 TG	870.7572	C22:3 ChE	720.6668
		C52:6 TG	868.7415	C22:4 ChE	718.6511
		C54:2 TG	904.8357	C22:5 ChE	716.6354
		C54:3 TG	902.8200	C22:6 ChE	714.6197
		C54:4 TG	900.8043	C24:6 ChE	742.6511
		C54:5 TG	898.7886		
		C54:6 TG	896.7729		
		C54:7 TG	894.7572		
		C54:8 TG	892.7415		
		C56:3 TG	930.8514		
		C56:4 TG	928.8357		
		C56:5 TG	926.8200		
		C56:6 TG	924.8043		
		C56:7 TG	922.7886		
		C56:8 TG	920.7729		
		C56:9 TG	918.7572		
		C58:4 TG	956.8671		
		C58:5 TG	954.8514		
		C58:6 TG	952.8357		
		C58:7 TG	950.8200		
		C58:8 TG	948.8043		
		C58:9 TG	946.7886		
		C58:10 TG	944.7729		
		C58:11 TG	942.7572		
		C60:8 TG	976.8357		
		C60:9 TG	974.8200		
		C60:10 TG	972.8043		
		C60:11 TG	970.7886		
		C60:12 TG	968.7729		
